# Evaluating Gene Fusions as Prognostic Biomarkers and Therapeutic Targets in Immune Checkpoint Blockade–Treated Advanced Melanoma: A Retrospective Analysis

**DOI:** 10.1158/2767-9764.CRC-25-0204

**Published:** 2025-08-13

**Authors:** Guadalupe Nibeyro, Verónica M. Baronetto, Agustín Nava, María R. Girotti, Laura Prato, Gabriel Morón, Elmer A. Fernández

**Affiliations:** 1 ScireLab, Fundación para el Progreso de la Medicina, Córdoba, Argentina.; 2 Consejo Nacional de Investigaciones Científicas y Técnicas (CONICET), Buenos Aires, Argentina.; 3 Facultad Regional Córdoba, Universidad Tecnológica Nacional, Córdoba, Argentina.; 4 Fundación Instituto Leloir-CONICET, Buenos Aires, Argentina.; 5 Research Department, Fundación Huésped, Buenos Aires, Argentina.; 6 Instituto de Tecnología (INTEC), Universidad Argentina de la Empresa (UADE), Buenos Aires, Argentina.; 7 Instituto Académico Pedagógico de Ciencias Básicas y Aplicadas, Universidad Nacional de Villa María, Córdoba, Argentina.; 8 Departamento de Bioquímica Clínica, Facultad de Ciencias Químicas, Universidad Nacional de Córdoba (UNC), Córdoba, Argentina.; 9 Centro de Investigaciones en Bioquímica Clínica e Inmunología (CIBICI), Consejo Nacional de Investigaciones Científicas y Técnicas (CONICET), Córdoba, Argentina.; 10 Facultad de Ciencias Exactas, Físicas y Naturales (FCEFyN), Universidad Nacional de Córdoba (UNC), Córdoba, Argentina.

## Abstract

Advanced melanoma, characterized by its aggressiveness and genomic complexity, demands improved prognostic and therapeutic strategies, particularly for patients with limited response to immune checkpoint blockade (ICB). Gene fusions, proposed as enhancers of tumor immunogenicity through neoantigens, also reflect chromosomal instability, which influences tumor evolution and therapy outcomes. However, their impact on melanoma remains unexplored. By retrospectively analyzing baseline tumors from 222 ICB-treated patients, we found a high tumor fusion burden (TFB-H) correlation with poor RECIST response, reduced overall survival (time-dependent ROC > 0.6, *P* << 0.01), and increased mortality risk (HR = 2, *P* < 0.01). TFB-H was found to be strongly associated with chromosomal instability (β = 0.72, *P* < 0.01), heightened proliferation, and diminished immune cytolytic activity. TFB-H was also linked to poor prognosis and immune impairment in nonadvanced melanoma tumors (*n* = 441) that have not received ICB treatment. These findings suggest that TFB-H tumors may exhibit an aggressive phenotype insensitive to ICB, probably due to immune evasion caused by intratumoral heterogeneity. Additionally, we identified targetable fusions, such as *KIAA1549::BRAF*, which represent therapeutic opportunities for advanced melanoma, including novel type II RAF inhibitors with potent activity against kinase fusions. Integrating gene fusion profiling into clinical practice may guide precision medicine strategies to overcome the limitations of ICB in advanced melanoma, offering prognostic insights and expanding therapeutic options, particularly with emerging fusion-specific inhibitors.

**Significance:**

The evidence of this work supports the idea that gene fusion profiling may serve as both a prognostic marker and a guide for alternative therapeutic strategies, including targeted fusion inhibitors, in patients less likely to benefit from ICB.

## Introduction

Melanoma, a type of tumor that typically arises from the skin because of the malignant transformation of melanocytes, is the leading cause of death among skin malignancies, with its incidence increasing worldwide (1). The major risk factor for melanoma is overexposure to UV radiation, which causes DNA damage and induces carcinogenesis (2). Once advanced melanoma metastasizes beyond its original site, whether to nearby tissues or distant organs, it becomes a life-threatening neoplasm with limited therapeutic options. However, immunotherapy has emerged as a cornerstone in the management of melanoma, offering significant advancements in patient outcomes. Additionally, for patients with specific genetic mutations, targeted therapies provide a complementary, precision-based treatment avenue. Despite recent advances in immunomodulatory therapies, which have increased median survival from approximately 6 months to nearly 6 years in advanced stages (3), some patients fail to respond, and others develop resistance over time (4). In this regard, significant efforts are still required to identify suitable biomarkers, both prognostic (i.e., that predict survival) and predictive (i.e., that predict response to therapy), which may help during treatment selection and monitoring (5).

The predominant genomic alterations in melanomas are single-nucleotide variants, which are currently used to make decisions for both targeted therapy and immunotherapy. In the context of immune checkpoint blockade (ICB) therapy, tumor mutation burden (TMB) has been associated with survival; however, this correlation is significant only for individuals with extreme TMB values, those in the top third and lower first quartiles, altogether accounting for only 50% of patients (6). Although TMB-derived neoantigens have been proposed as prognostic biomarkers in advanced melanoma, their predictive value remains controversial (7). In this sense, gene fusions have also been suggested as a potential source of neoantigens (8). However, a pan-cancer study found no significant association between predicted tumor fusion–derived neoantigens and outcomes of ICB therapy (9).

Although uncommon in melanoma, gene fusions (i.e., common cancer-driving rearrangements with a significant role in cancer progression) have been recognized as drivers in certain melanomas and identified as key therapeutic targets (1, 10). Patients with gene fusions matched to FDA-approved targeted therapies have shown high response rates. This observation supports the potential therapeutic benefit of gene fusions (11), even following immunotherapy progression (12).

Melanoma progression involves complex genomic rearrangements. Numerous structural alterations, including gene fusions, have been identified through spectral karyotyping in melanoma cell line studies and through next-generation sequencing in late-stage metastatic melanoma (13, 14). The emergence of gene fusions is often linked to chromosomal instability (CIN; ref. 15), a critical factor driving tumor progression, metastasis, and drug resistance (16, 17). CIN not only drives tumor evolution but also is associated with poor prognosis across multiple cancer types. Additionally, CIN promotes intratumor heterogeneity (ITH; ref. 18), which has been shown to impair immune responses (19, 20) and is associated with poor outcomes following T-cell checkpoint blockade therapy in advanced melanoma (19, 21).

Therefore, based on the accurate and cost-effective identification of gene fusions using current RNA sequencing (RNA-seq) technology and the demonstrated improvements in matched gene fusion–targeted therapy outcomes (11), we hypothesize that characterization and quantification of gene fusions in ICB-treated advanced melanoma could hold significant clinical relevance. Moreover, because gene fusions may arise as a by-product of CIN (22), tumor fusion burden (TFB) could potentially act as a prognostic biomarker for immunotherapy outcomes. This assumption is further supported by CIN association with both the high proliferative potential, a well-established factor influencing immunotherapy efficacy (23), and the ITH resulting from CIN. More importantly, the ease of detecting gene fusions and their straightforward implementation in clinical settings represent a key advantage in the potential utility of gene fusions in precision oncology.

The association between TFB and ICB outcomes remains underexplored in advanced melanoma. The Cancer Genome Atlas (TCGA) network previously reported an average of approximately two gene fusions per patient. The calculation included melanomas from both early and advanced stages, rather than being focused on advanced melanoma (24). TFB has been investigated in non–ICB-treated solid tumors, yielding controversial results. For instance, Wagle and colleagues (25) reported that high TFB correlated with increased immunogenicity in prostate cancer, suggesting that patients with high TFB (TFB-H) may be suitable candidates for ICB therapy. Conversely, studies in head and neck squamous cell carcinoma (26) and gastric cancer (27) indicated that low TFB (TFB-L) tumors might be better suited for immunotherapeutic approaches. This controversy underscores the importance of exploring the TFB–ICB outcome relationship specifically in advanced melanoma.

**Figure 1:**
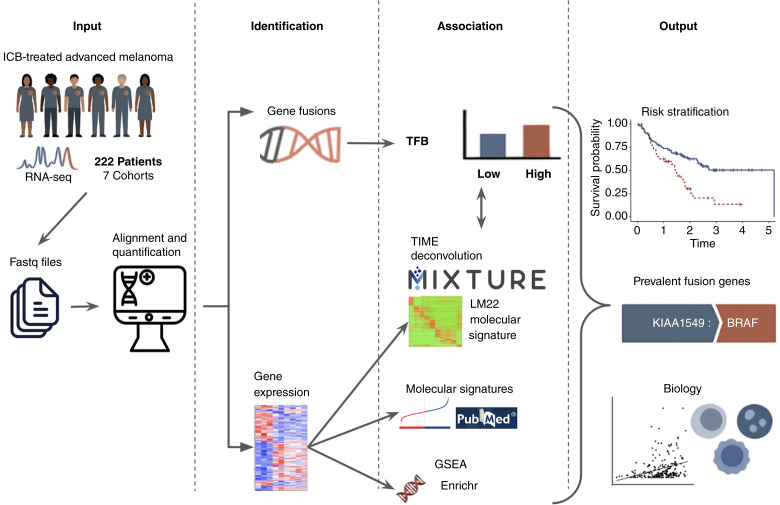
Analytic workflow. GSEA, gene set enrichment analysis.

Moran and colleagues (1) studied therapeutic implications of gene fusions in melanoma; however, the authors included very few advanced melanoma patients receiving immunotherapy but did not include outcome information. Moreover, they used targeted sequencing techniques designed to detect specific fusion transcripts involving genes commonly rearranged in solid tumors, limiting the ability to characterize the complete gene fusion repertoire. The mentioned earlier studies emphasize the growing interest in understanding the role of gene fusions; yet, none has specifically focused on advanced melanoma cohorts with ICB outcome information, as we propose in this study. In this study, we investigate the relationship between TFB, CIN, and ICB outcomes in advanced melanoma by integrating data from multiple ICB-treated melanoma cohorts. We also devise the complete repertoire of high-confidence gene fusions through baseline RNA-seq tumor samples, aiming to address both, a prognostic factor by identifying the total number of gene fusions per patient and potential therapeutic targets by recognizing candidate driver fusions. Figure 1 depicts the analytic workflow.

## Materials and Methods

### Collection of data from ICB cohorts

Seven public advanced melanoma cohorts with ICB outcome information were analyzed. Raw RNA-seq data were obtained from the European Nucleotide Archive (ENA). Metadata from the original publication were kept. Only pre–ICB treatment biopsy samples were used. Thus, a total of 222 patients were included: 73 from Gide and colleagues (28); 25 from Hugo and colleagues (6); the Snyder and colleagues cohort (29), which only had seven publicly available RNA-seq datasets; the MGH dataset, holding 17 patients (30, 31); and 25 patients from Riaz and colleagues (32). The latter cohort also comprises patients who progressed to ipilimumab and who were excluded from the analyzed cohort because at the time of biopsy they were already exposed to ICBs. Overall survival (OS) information was available for the five cohorts. Two additional cohorts were incorporated, 064 and 067, with a total of 42 and 33 patients, respectively, only for genomic profile validation purposes, because the event related to OS was not available for these patients (33). Patients from the Gide cohort were analyzed for *BRAF* mutation and treated with *BRAF* inhibitors when positive (*N* = 17) prior to ICB treatment; 12 patients from the Hugo cohort, who were positive for *BRAF*, *NRAS*, or *NF1* mutations, were treated with MAPK inhibitors. Finally, the Snyder study refers to a previous treatment with IL-2 and cytotoxic chemotherapy in some patients. Due to the cross-resistance between tyrosine kinase inhibitors (TKI) and ICBs (34, 35), as well as the heterogeneity of available ICB databases, the cohorts were analyzed separately. However, all the analyzed cohorts are characterized by advanced melanomas.

Table 1 shows the main characteristics of each cohort (see Supplementary Table S1 for further cohort information).

**Table 1:** Relevant characteristics of the analyzed cohorts (222 patients)

​	Gide (28)	Hugo (6)	Riaz (32)	Snyder (29)	MGH (30, 31)	CheckMate 064 (33)	CheckMate 067 (33)
ICB	Anti–PD-1 Anti–PD-1 + anti-CTLA4	Anti–PD-1	Anti–PD-1	Anti-CTLA4	Anti–PD-1Anti–PD-L1Anti-CTLA4	Anti–PD-1Anti-CTLA4	Anti–PD-1Anti-CTLA4
TX	Nivolumab, pembrolizumab, and ipilimumab	Nivolumab and pembrolizumab	Nivolumab	Ipilimumab	—	Nivolumab Ipilimumab	Nivolumab Ipilimumab
Previous TX	BRAF inhibitor	MAPK inhibitor	—	IL-2, cytotoxic chemotherapy	—	—	—
Subjects	73	25	25	7	17	42	33
OS data	Yes	Yes	Yes	Yes	Yes	No	No
ENA Nº	PRJEB23709	PRJNA312948	PRJNA356761	PRJNA693857	PRJNA706446PRJNA476140	PRJNA923698	PRJNA923698

### Non-ICB melanoma validation cohort

One additional melanoma cohort, described by Badal and colleagues (36), with raw RNA-seq data from 51 tumors consisting of primary melanomas of the skin, was acquired from the ENA browser (PRJNA385075). Tumors were collected from treatment-naïve patients, and none of them presented stage IV melanoma. Information on OS outcome was available for 44/51 samples, but it was not related to immunotherapy.

### RNA-seq processing and fusion detection

Raw RNA-seq data were trimmed with Trim-Galore v0.6.6 (RRID: SCR_011847) and aligned to the reference genome hg38 with STAR v2.7.10b (RRID: SCR_004463). The aligned BAM file was used to assess gene expression with the R-based FeatureCounts function (RRID: SCR_012919). Gene fusions were detected from the aligned bam file with Arriba v2.3.0 (RRID: SCR_025854; see Supporting Information).

### TCGA cohorts

Gene fusions from skin cutaneous melanoma (SKCM) were collected from the TCGA-SKCM cohort from Gao and colleagues (37). Clinical information was retrieved through TCGAbiolinks (RRID: SCR_017683). Stage IV melanomas and samples with no reported stage information were excluded, resulting in 95 pretreatment primary tumor biopsy samples (identified by “01” in the sample type code). Additionally, 295 biopsies from the site of metastases (“06” type code) were analyzed separately. Transcript per million (TPM) expression matrices were obtained from Rocha and colleagues (38).

### TFB

TFB was defined as the total high-confidence gene fusions detected per subject by Arriba software. This confidence tag provided by the software reflects three main aspects, according to the Arriba documentation: the transcript is aberrant (nonexistent in healthy tissue), it can be explained by an underlying genomic rearrangement, and it is not an artifact (39). The transcripts that did not have a protein product were also retained because it was proven that fusion RNA transcripts may act as long noncoding RNAs with transcriptional regulation capabilities (40). Fusions between the same pair of genes but with different breakpoints were considered separately (as different fusions) because genomic instability could lead to the different rearrangements (Supplementary Table S2).

In TCGA cohorts, TFB represents the number of fusions detected per patient by Gao and colleagues (37), because the raw data were not publicly available. Differences in the fusion detection pipeline used for TCGA, compared with that implemented for ICB cohorts, could affect the distributional characteristics of TFB.

### Molecular signatures

Gene signatures were used with gene symbols when available. Genes without a valid gene name were discarded. Signature scores for proliferation (41), cytolytic activity (19), and centrosome amplification (42) were calculated according to the original publications from TPM expression matrices. A linear model was applied to find a correlation with TFB.

### Treatment outcome assessment

RECIST v1.1 is the standard for response assessment in oncology; however, it has been found controversial for immunotherapy due to the unique response patterns exhibited by ICB-treated patients, which is associated with T-cell activation mechanisms (43–45). As a result, the RECIST working group developed iRECIST specifically for use in cancer immunotherapy trials. However, it was not possible to use this standard in this work because iRECIST response criteria were not available for the evaluated cohorts. There is no consensus on how to define responders versus nonresponders to ICB therapy based on RECIST criteria; patients with stable disease are considered responders, nonresponders, or even excluded in different studies (28, 32). Therefore, ICB therapy outcome was evaluated through OS, which is the ultimate endpoint (44).

The threshold to classify patients into TFB-L vs. TFB-H groups was selected based on the TFB (number of fusions) showing a statistically significant difference in the OS Kaplan–Meier (KM) curve of the Gide cohort (as the discovery set), after performing a systematic scan of thresholds and *P* values across all possible cutoffs. At the same time, when the selected threshold is applied to classify patients in the validation cohorts, each TFB group should have at least 10% of patients. Notably, the selected threshold corresponds to the third quartile of the TFB distribution across all cohorts, a commonly used and data-driven cutoff in KM analyses, thus avoiding arbitrary dichotomization. Then, OS was studied separately on each validation cohort, through KM curves, comparing TFB groups. One patient from the Gide cohort died of non–melanoma-related causes; therefore, he was left out from the OS analysis but incorporated for subsequent exploration and was assigned to the TFB-H or TFB-L group according to the calculated threshold. The association between TFB and OS was not analyzed in the Snyder cohort because of the small number of patients. To assess potential confounding factors, such as cohort characteristics and prior treatments, a multivariate Cox regression analysis was performed for TFB considering it both as a continuous and a dichotomous variable. The corresponding concordance index was also calculated. Additionally, a 3-year time-dependent ROC curve was evaluated. To validate the robustness of the AUC result, given the limited sample size, a bootstrap method with 100 iterations was applied using sampling with replacement. The median AUC across all iterations was calculated and tested for significance against the null value of 0.5.

The threshold calculated for the Gide discovery cohort was also used to classify non-ICB cohort patients into TFB-H and TFB-L groups.

### Tumor immune cell infiltrate

FeatureCounts results were transformed into TPM, and tumor immune cell infiltration proportion was calculated with the state-of-the-art tumor microenvironment (TME) deconvolution algorithm, MIXTURE, using the LM22 molecular signature covering 22 human immune cell phenotypes (46).

### Differential expression and gene set enrichment analysis

Differential expression analysis was performed in each cohort separately to dig into the biological processes and pathways of the defined TFB groups (TFB-H/TFB-L). FeatureCounts results were transformed into counts per million. The scaling factors for library sizes in the count matrix were calculated with the trimmed mean of M values (TMM) method. The differential gene expression analysis was performed using the voom function from the limma package (RRID: SCR_010943), with a log fold change = 0.1 (47).

Gene set enrichment analysis was performed with EnrichR (48) using the differentially expressed genes and the EnrichR r-package CRAN tool (RRID: SCR_001575; ref. 49) with six databases: “GO_Molecular_Function_2023,” “GO_Cellular_Component_2023,” “GO_Biological_Process_2023,” “Reactome_2022,” “KEGG_2021_Human,” and “MSigDB_Hallmark_2020.” Then, only those gene sets present in at least four of seven cohorts were kept, aiming to find a phenotype that consistently manifests in all cohorts, as described in previous studies (50, 51).

### Prevalence of fusion genes

The prevalence of fusion genes was defined, for each gene, as the number of patients having that gene involved in different fusions independently of the fusion partner. The prevalence of each gene was calculated in the TFB-H and TFB-L groups.

### Statistical analysis

All the statistical analyses and the plots were performed in R v4.2.0 (RRID: SCR_001905) with ggplot2 (RRID: SCR_014601), ggpubR (RRID: SCR_021139), epiR (RRID: SCR_021673), smoothROCtime, survival (RRID: SCR_021137), and survminer (RRID: SCR_021094) libraries.

### Data availability

The data presented in this study are available in the ENA, with reference numbers PRJEB23709, PRJNA312948, PRJNA356761, PRJNA693857, PRJNA706446, PRJNA476140, PRJNA923698, and PRJNA385075. A GitHub repository was also created to ensure reproducibility and is publicly available at https://github.com/gnibeyro/Gene_Fusion_ICB. Other data generated in this study are available upon request from the corresponding author.

## Results

### TFB is strongly correlated with CIN, impaired immune response, and cell proliferation

**Figure 2:**
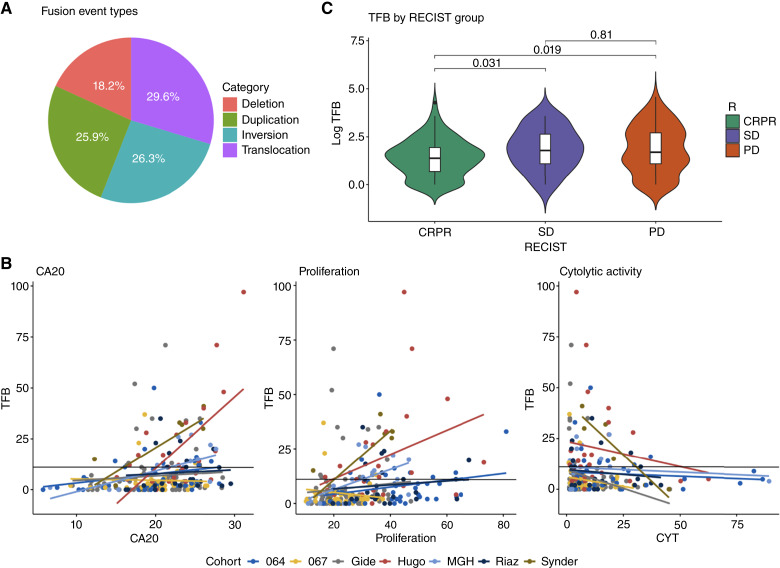
**A,** Fusion event type percentages. **B,** Linear models assessing the association between TFB and each of the molecular signature scores by cohort, including chromosomal instability score, cytolytic activity (CYT) score, and proliferation score. **C,** TFB distribution by RECIST groups. CRPR, complete response or partial response; PD, progressive disease; SD, stable disease.

The distribution of TFB (i.e., the total number of high-confidence gene fusions per patient) over the different cohorts ranged between 0 and 97, with a median of 4 (Supplementary Table S1). The fusion events can be classified into four main structural types: deletions (18.2%), duplications (25.9%), inversions (26.3%), and translocations (29.6%), as shown in Fig. 2A. The first three corresponds to intrachromosomal rearrangements (70.4%) and translocations to interchromosomal rearrangements (29.6%). We wondered whether the observed TFB could be attributed to CIN and whether it correlated with immune response and cell proliferation. Linear models relating the TFB to each molecular signature were evaluated using previously well-documented molecular signatures for CIN (CA20, a centrosome amplification score; ref. 42), immune status (the cytolytic score; ref. 19), and proliferation (41). Figure 2B shows the linear association between TFB and the different scores achieved by the three different molecular signatures. CIN was significantly associated with increased TFB (*β* = 0.72, *P* < 0.01), whereas TFB exhibited a significant and negative association with the cytolytic score (*β* = −0.14, *P* < 0.05). We found a significant and positive association between a molecular signature of proliferation and TFB (β = 0.25, *P* < 0.01; the linear models are provided in Supporting Information); this result is consistent with evidence linking advanced tumor aggressiveness to uncontrolled proliferation (52).

### TFB-H is associated with the worst ICB outcome

Because gene fusions are reliably and efficiently identified through RNA-seq, and considering the established links between the observed TFB with CIN and the consequent cell proliferation and immune impairment, we investigated whether TFB might also be associated with immunotherapy response. Despite the debated nature of the RECIST criteria, we observed that patients with stable or progressive disease had a statistically significant higher TFB than responders (those with complete or partial response; Fig. 2C).

**Figure 3:**
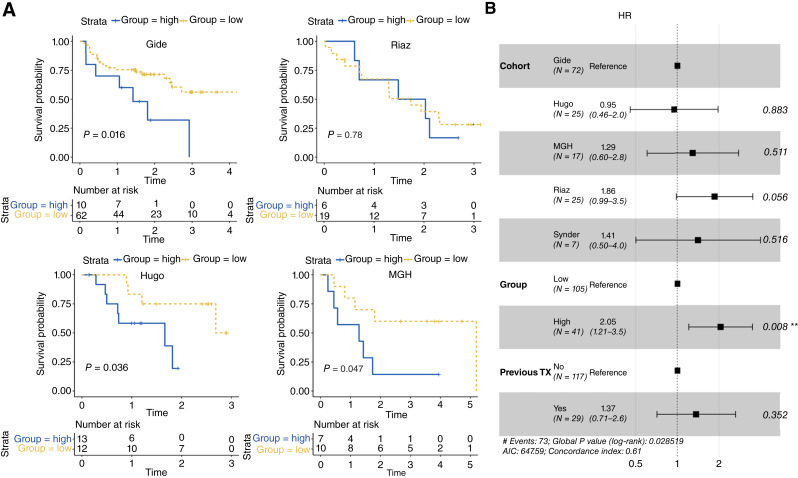
**A,** KM OS curves by high/low-TFB groups for Gide discovery cohort; Riaz validation cohort; Hugo validation cohort; and MGH validation cohort. **B,** Forest Plot for Cox proportional hazards model.

To gain a deeper insight into these findings, we used OS as a surrogate outcome measure. Initially, using the Gide cohort as the discovery set, we performed a KM survival analysis classifying the subjects into patients carrying either a high or low number of TFB (TFB-H/TFB-L groups, respectively) by spanning, significantly OS-associated, different TFB values. The chosen TFB classification value resulted in TFB = 11, satisfying that all the validation cohorts should have at least 10% of patients in each TFB group, as in previous approaches (Supplementary Table S3; ref. 53). The TFB-H group (subjects with more than 11 high-confidence fusions) was found to be significantly associated with poor OS (*P* = 0.016). Subsequently, a survival analysis was performed by dividing patients from the validation cohorts into TFB-H or TFB-L groups based on the selected threshold (TFB > 11). TFB was consistently associated with OS, with the TFB-H group being linked to short survival in all validation cohorts, except in the Riaz cohort, in which no significant association was observed (Fig. 3A). Then, to explore the factors influencing the association between TFB and outcomes, we performed Cox regression analyses for each clinical variable available and TFB independently. Neither the source cohort nor prior TKI treatment showed a significant association with outcomes, whereas TFB did (see Supplementary information). Additionally, time-dependent ROC analysis demonstrated a significant predictive performance of TFB at 3 years (AUC = 0.61, bootstrap Wilcoxon test, *P* < 2.2e−16; ref. 54). Finally, a multivariate cox regression model analysis was performed, using TFB as dichotomized variable (TFB-H vs. TFB-L) and adjusting for the available potential confounders (i.e., source cohort and prior TKI treatment). The results show that the TFB-H group presents a twofold increase in event risk (HR = 2.05, *P* = 0.008), with a concordance index of 0.61. This model identified TFB-H as the only significant predictor affecting OS (Fig. 3B). When using TFB as a continuous variable, the high risk remains statistically significant, but the HR is lower than using TFB groups.

Finally, Cox regression models including TFB groups were also performed, adjusting for *PD-L1 (CD274)* gene expression (log-transformed TPM) and ICB treatment regimens. The TFB-H group remained significantly associated with worse OS (see Supplementary Material).

### The TFB-H group is enriched with M2 macrophages

**Figure 4:**
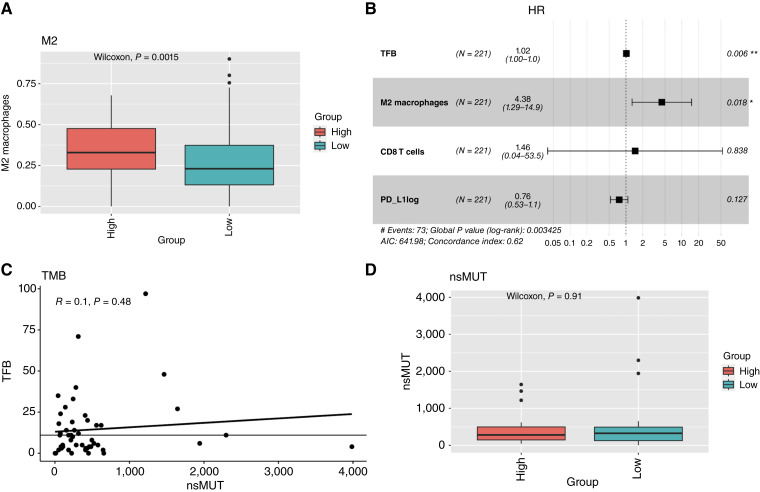
**A,** M2 proportion distribution by TFB groups. **B,** Forest plot for Cox proportional hazards model. **C,** linear model correlation between TFB and nonsynonymous mutations (nsMUT). **D,** nsMUT distribution by TFB groups.

Considering that the success of ICB therapy depends on the tumor-immune microenvironment, we analyzed immune cell proportions according to TFB group classification. A higher proportion of M2 macrophages, known for their immunosuppressive and protumor activity, was observed in the TFB-H group compared with the TFB-L group (Fig. 4A). To determine whether the increased risk observed in TFB-H patients was driven by fusion burden itself or by TME factors, we performed a multivariate Cox model incorporating M2 macrophage and CD8^+^ T-cell proportions along with *PD-L1* expression. In this analysis, only TFB and M2 macrophages were independently associated with survival risk (Fig. 4B).

Finally, TMB was assessed in the Hugo and Riaz cohorts (the only datasets with available TMB data). No statistically significant correlation was found between TMB and TFB (Fig. 4C), and there was no significant difference in TMB between TFB-H and TFB-L groups (Fig. 4D).

### Gene expression also reveals proliferative phenotypes for the TFB-H group in ICB cohorts

To investigate potential biological correlates of TFB levels that might explain TFB association with survival following ICB treatment, gene set enrichment analysis was performed independently for all ICB cohorts, comparing low and high TFB groups. Subsequently, pathways dysregulated in more than 50% of the cohorts were studied with the aim to find biological coherence among cohorts. This analysis identified five upregulated and one downregulated pathways in the TFB-H group (Supplementary Table S4).

The regulation of the cell adhesion pathway, which is related to the invasive potential (55), was found to be downregulated in the TFB-H group. In contrast, several cell cycle–related pathways (cell cycle, mitotic cell cycle, regulation of mitotic cell cycle phase transition, and E2F targets) were upregulated in the TFB-H group, as expected from the previous proliferation score analysis. The E2F, a key transcription factor for proliferation, is associated with replication stress, a common cause of CIN (56).

### TFB is also prognostic on non–ICB-treated, nonadvanced melanoma with immune impairment association

Our findings suggest that TFB-H would be associated with poor outcomes following ICB treatment in advanced melanoma. This unfavorable prognosis suggests a more aggressive tumor phenotype and a less active immune response rather than increased immunogenicity driven by new neoantigens. As a next step, we aimed to explore TFB-associated behavior in nonadvanced melanomas and assess its impact independently of subsequent treatments.

**Figure 5:**
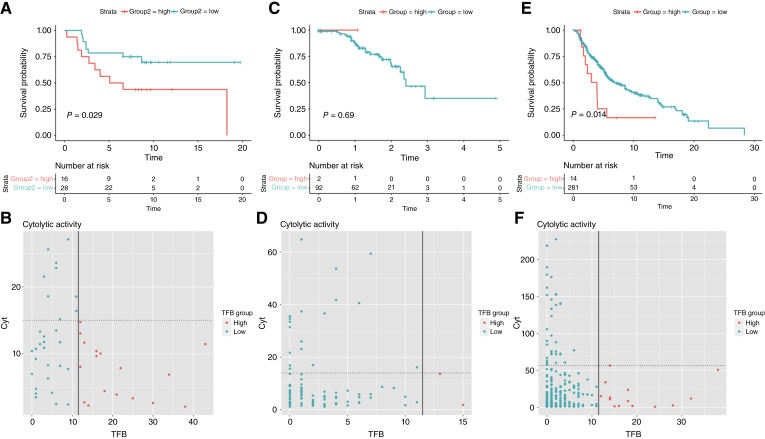
**A,** KM curve for OS by TFB status in the Badal cohort. **B,** Scatter plot showing the relationship between CIN (*Y*-axis) and TFB (*X*-axis) for the Badal cohort. **C,** KM curve for OS by TFB status in the TCGA-01 cohort. **D,** Scatter plot showing the relationship between CIN and TFB for the TCGA-01 cohort. **E,** KM curve for OS by TFB status in the TCGA-06 cohort. **F,** Scatter plot showing the relationship between CIN and TFB for the TCGA-06 cohort.

First, the Badal cohort was analyzed, and TFB was calculated for all 51 subjects, who were then classified as TFB-H or TFB-L based on the same TFB threshold established in the Gide cohort (TFB > 11). The KM curves showed that in this dataset, TFB-H patients had worse OS than TFB-L patients (*P* < 0.05), although they did not have advanced disease nor did they receive ICB treatment (Fig. 5A). Next, molecular signatures were analyzed. In these earlier stages, CIN was not associated with TFB; however, the cytolytic score differed significantly between TFB-H and TFB-L groups (Wilcoxon test, *P* < 0.05), with TFB-H seeming to have reduced immune system activity. The scatter plot in Fig. 5B displays the relationship between cytolytic activity and TFB, with the data points showing that the highest values of cytolytic activity correspond to the TFB-L group.

Then we attempted to validate these findings in 95 primary tumor biopsies from the SKCM-TCGA-01 cohort. The patients were classified into TFB groups based on the adopted threshold of 11. Because gene fusion identification was conducted by Gao and colleagues (37), TFB distribution was different from that of the other cohorts; thus, the TFB-H group had only two patients. In this scenario, the KM curves resulted inadequate, but both patients died within the first year (Fig. 5C); in addition, the two TFB-H patients had low values of cytolytic scores (Fig. 5D).

Finally, we analyzed 295 additional subjects with metastatic site biopsy samples (SKCM-TCGA-06 cohort). Figure 5E shows that TFB-H was also associated with worse outcomes (*P* < 0.05) than TFB-L. The analysis of the molecular signatures showed that CIN was not correlated to TFB. Lastly, TFB-H subjects present low cytolytic scores, in line with the observed immune impairment across cohorts (Fig. 5F).

### Association of fused gene prevalence with TFB groups

**Figure 6:**
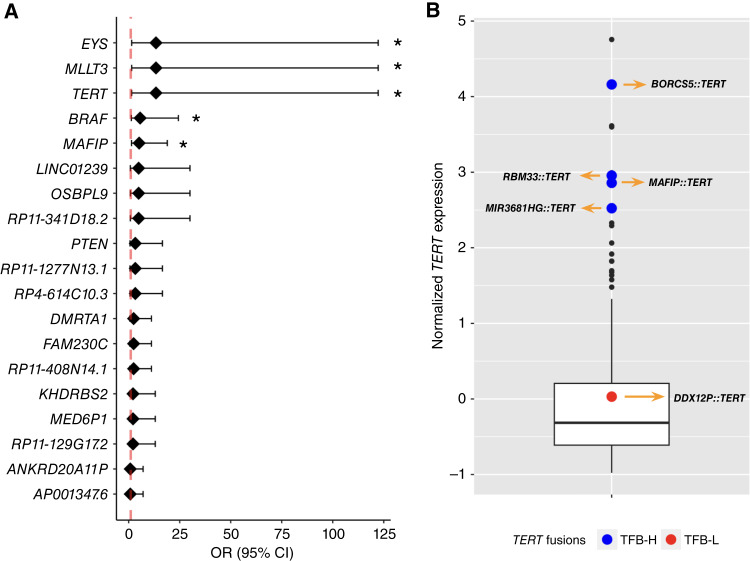
**A,** OR of prevalent genes associated with TFB-H; *, statistically significant; *P* value, *χ*^2^ significance test. CI, confidence interval. **B,** Box plot showing the distribution of *z*-score log (TPM + 1) expression of *TERT* gene. Red and blue dots correspond to samples with *TERT* fusions.

The association between genomic instability and gene fusions has been previously reported, and the possibility that nonrecurrent gene fusions may function as passenger events has been suggested (22, 57). In our studied cohorts, we did not observe common fusions, which is consistent with that suggestion. However, gene fusions are often reported, with an emphasis on one of the fused genes (as seen in *BRAF* or *NTRK* fusions). Therefore, we explored whether certain genes might be more frequently involved within specific TFB groups rather than focusing solely on the prevalence of specific gene fusions. This approach is particularly relevant because constitutive gene activity can arise not only from mutations but also from gene fusions, as demonstrated for *BRAF* fusions in various solid tumors, including melanoma (10). Six prevalent genes were significantly more likely to be fused in the TFB-H group than in the TFB-L group (*χ*^2^ test, *P* < 0.05), which is associated with poor prognosis, and were rarely observed in the TFB-L group (Fig. 6A). *MYO10* was found exclusively in the TFB-H group (9.26% of TFB-H subjects), whereas the rest (*MLLT3*, *TERT*, *EYS*, *MAFIP*, and *BRAF*) were present in at least 7% of TFB-H subjects (Supplementary Table S5). In the TCGA-06 cohort, *MLLT3* was found as fusion partner in two patients (one TFB-H and one TFB-L), *BRAF* in three patients (one TFB-H and two TFB-L), and *MYO10* in seven patients (six TFB-H and one TFB-L), demonstrating the presence of prevalent genes in metastatic sites.

### Identifying fused genes with oncogenic potential

Among the prevalent gene fusions, we focused on those involving *TERT* and *BRAF,* whose fusions have been previously described as oncogenic with an already elucidated pathogenic mechanism. *TERT* fusions are gaining attention as potential therapeutic targets, and it was previously reported in the metastasis of a patient with advanced melanoma without immunotherapy (1). Genomic mutations in *TERT* have been experimentally validated as drivers of oncogenesis, particularly in melanoma (1, 58). These mutations increase transcript levels of *TERT*, which is typically not expressed in most cells. The resulting elevated telomerase activity is associated with rapid tumor growth and poor prognostic factors, such as a heightened risk of metastasis. Consequently, *TERT* inhibition represents a promising therapeutic strategy for melanoma (59). Figure 6B illustrates increased *TERT* expression in tumors harboring *TERT* fusions, as previously reported (60), suggesting that *TERT* fusions may represent an alternative mechanism for telomerase activation in advanced melanomas, potentially functioning as a driver fusion.

The clinical significance of *BRAF* fusions was established, and it became even more important with the emergence of novel type II RAF inhibitors, which has been shown to potently inhibit *BRAF* fusions. Well-known *BRAF* fusions were identified in eight patients across the analyzed cohorts, including a recurrent fusion, *AGK::BRAF* (*n* = 2). Notably, this fusion was also detected in a patient from the TCGA-06 cohort. This finding supports the potential role of *AGK::BRAF* as a driver, as it was identified in a biopsy from a metastasis. Several of these fusions, such as the mentioned *AGK::BRAF*, *KIAA1549::BRAF*, and *MKRN1::BRAF*, have been previously reported in melanoma and other solid tumors. These fusions demonstrate oncogenic potential and responsiveness to treatment with TKIs (61–65) and are currently used to guide targeted therapies. It is known that in cases in which *BRAF* mutations are absent, *BRAF* fusions may gain TK function, becoming oncogenic drivers. The mechanism to explain the TK activation is a truncation-mediated loss of the inhibitory domains within the N-terminus of the *BRAF* protein, offering a therapeutic option (62). In this study, three *BRAF* fusions sharing the same *BRAF* breakpoint as that of *KIAA1549::BRAF* were found in three subjects, with *MLANA*, *MAD1L1*, and *MKRN1* as gene partners. Because the mentioned gene fusions share the *BRAF* breakpoint, they might exhibit the same biological behavior, thus providing relevant information to the clinician. Indeed, recent studies suggest improved survival when immunotherapy is continued until disease progression, followed by *BRAF/MEK* inhibition (12); this strategy may be particularly relevant, given the poor OS in the TFB-H group after ICB treatment. The identification of *BRAF* fusions highlights the clinical significance of gene fusions in advanced melanoma.

## Discussion

Our study demonstrates the feasibility and clinical relevance of gene fusion identification and quantification using baseline RNA-seq data in patients with advanced melanoma, particularly in relation to ICB outcomes. Melanoma remains a prevalent malignancy with limited therapeutic options and a lack of reliable prognostic biomarkers in advanced stages. Our findings highlight that assessing TFB on a per-patient basis provides valuable insights into tumor evolution and prognosis, whereas the accurate detection of both known and novel gene fusions uncovers new therapeutic opportunities. Improved outcomes in patients with gene fusions matched to targeted therapies were already reported by Nikanjam and colleagues (11).

Gene fusions have been widely studied in cancer as key contributors to tumor evolution (66), arising as by-products of CIN, which is linked to tumor progression. In the context of immunotherapy, they are recognized for generating neoantigens that can enhance immune responses (8). However, no association has been reported between predicted tumor fusion–derived neoantigens and ICB outcomes (9). Whereas the impact of TFB on the immune TME has been explored, most studies have focused on non–ICB-treated patients, leading to controversy about its clinical relevance (25–27). In advanced melanoma, ICB outcomes have been extensively investigated using various omics approaches; yet, the role of gene fusions remains largely unexplored (6). Early studies on gene fusion detection in advanced melanoma relied on targeted sequencing of a limited set of commonly rearranged genes in solid tumors, often involving very few ICB-treated patients and lacking OS data (1). Other efforts, such as the TCGA study, analyzed multiple melanoma stages but did not focus on ICB-treated patients (24). Our study is the first to comprehensively characterize the full gene fusion repertoire and evaluate its association with ICB outcomes in a large cohort of patients with advanced melanoma.

In this study, we observed a higher risk of poor outcomes following ICB treatment in TFB-H subjects than in patients with a low number of fusions, a result that underscores the limited impact of neoantigens. The neoantigens generated from gene fusions may be insufficient to counteract the high aggressiveness of TFB-H tumors, suggesting that these tumors remain highly aggressive despite the presence of new immune targets. These results are in line with previous studies, in which tumors such as head and neck squamous cell carcinoma and gastric cancer, typically classified as “hot” tumors because of their high TMB and inflamed TME, have shown that patients with high TFB exhibit reduced immune activity and poor prognosis, aligning with our findings in melanoma (9, 26, 27, 67, 68). However, it has also been shown that the immunologic impact of TFB may be highly context-dependent. In prostate adenocarcinoma (PRAD), a “cold” tumor characterized by low immune infiltration and low TMB, relatively high levels of gene fusions have been observed. Yet, previous studies suggest that a subset of PRAD tumors with elevated TFB may exhibit features of immune activation and could potentially benefit from ICB (9, 25, 67, 68). This suggests that the role of gene fusions in shaping tumor immunogenicity may differ by tumor type. In immune-hot tumors, high TFB may reflect increased CIN and immune escape mechanisms. In contrast, in immune-cold tumors, high TFB might occasionally generate novel neoepitopes and increase immune visibility, though this remains to be further validated.

In this context, TFB strongly correlates with CIN in advanced melanomas. Gene fusions arise as by-products of chromosomal structural alterations and are closely linked to CIN. CIN has long been recognized as a hallmark of clinically aggressive cancers, and our findings demonstrate that poor outcomes in TFB-H patients persist despite ICB therapy. This result is supported by the diminished immune system activity found in TFB-H subjects. The observed clinical behavior may be attributed to the association between CIN and increased ITH, which enables subpopulations of cells to develop more aggressive phenotypes, as evidenced by their high proliferation rates observed in this study. This heterogeneity dilutes reactive neoantigens, weakens antitumor immunity, and ultimately reduces the efficacy of ICB therapies (19, 52).

A deeper characterization of the TME over the TFB groups demonstrates that TFB-H patients presented an increment of M2 protumoral macrophages, an observation that has been previously described, in which tumors with high CIN promote an immunosuppressive TME characterized by suppressive macrophages and dysfunctional T cells. Mechanistically, CIN might activate the cGAS-STING pathway in a noncanonical way, favoring endoplasmic reticulum stress responses over IFN signaling, thereby suppressing antitumor immunity (69). These findings suggest that TFB-H tumors may exhibit primary resistance to ICB, potentially because of their association with an immunosuppressive microenvironment driven by CIN.

The results were then compared with TFB behavior in patients with nonadvanced melanoma without ICB treatment outcomes. TFB was associated with OS outcomes regardless of therapy or disease stage. In this context, TFB-H patients exhibited reduced immune system activity compared with TFB-L patients, suggesting that TFB-L patients may be better candidates for immunotherapy, likely because of greater immune system integrity. Additionally, the observed association between CIN and TFB in advanced melanoma was not detected in nonadvanced stages, suggesting that this correlation emerges during tumor progression. Indeed, copy-number alterations and other CIN-associated features are uncommon in nevi and early melanoma but become prevalent in invasive and metastatic stages, suggesting a stepwise transition from UV-induced mutagenesis to CIN-driven tumor evolution (20, 70). In advanced melanomas, slow proliferation in TFB-L subjects also seems to enhance ICB efficacy. These findings highlight TFB as a potential prognostic tool and underscore the need for new strategies to effectively target TFB-H tumors.

In this scenario, identifying well-known gene fusions, such as those involving *BRAF* or *TERT*, previously reported as oncogenic drivers, provides valuable clinical insights into targeted therapy. This potential is further supported by the ongoing development of novel type II RAF inhibitors, which demonstrate potent activity against specific kinase fusions and are currently being evaluated in clinical trials (71). These inhibitors could be used in combination with ICB or as a subsequent therapy following disease progression (1, 12).

These findings have important implications for precision medicine, potentially guiding more tailored therapeutic strategies and enhancing the clinical relevance of gene fusion identification and quantification in patients with advanced melanoma.

### Limitations

Our search recognized a limited number of publicly available RNA-seq datasets of ICB-treated advanced melanoma samples. Public cohort analyses are often constrained by limited metadata, which restricts the available covariates for study. Despite this, we provide a comprehensive overview of our bioinformatics pipeline (Supplementary Material), which enables future application of our approach to these and newly emerging cohorts. Validation in larger cohorts and prospective clinical trials is necessary. Increasing the availability of public databases with transcriptomic data from clinical trials involving various immune therapies with known responses and outcomes is crucial for advancing our understanding of the complex interactions between genomic instability, neoantigen generation, and immunomodulatory therapies. Expanding access to such datasets will inform the clinical management of patients with cancer receiving immunotherapy and may help address critical gaps in our understanding of cancer immunity, which require further investigation (72).

## Supplementary Material

Supporting_Information “Bioinformatic pipeline”, analysis workflow. “Linear model for molecular signatures”. “Univariate Cox regression analysis”. “Multivariate Cox regression analysis”. “Linear model for tumor microenvironment”.

Table S1 “Characteristics of patients”

Table_S2 Fusions detected.

Table_S3 Number of patients from each cohort with Low/High TFB.

Table_S4 Dysregulated pathways.

Table_S5 Gene appearance frequencies between High vs Low TFB groups, ordered by OR significance.
