# Supplementary material for: Evaluating Gene Fusions as Prognostic Biomarkers and Therapeutic Targets in Immune Checkpoint Blockade–Treated Advanced Melanoma: A Retrospective Analysis
Source: Cancer Res Commun. 2025 Aug 13;5(8):1332–43. doi: 10.1158/2767-9764.CRC-25-0204 (PMC12344778; doi:10.1158/2767-9764.CRC-25-0204)
Supplement: Supporting_Information — “Bioinformatic pipeline”, analysis workflow. “Linear model for molecular signatures”. “Univariate Cox regression analysis”. “Multivariate Cox regression analysis”. “Linear model for tumor microenvironment”. [file crc-25-0204_supporting_information_suppsi.pdf]

Supporting Information

Evaluating Gene Fusions as Prognostic Biomarkers and Therapeutic Targets in Immune Checkpoint Blockade-Treated Advanced Melanoma: a Retrospective Analysis.

Guadalupe Nibeyro[1,2,3], Verónica M. Baronetto[1,2], Agustín Nava[2,4,5], María R. Girotti[6], Laura B. Prato[7], Gabriel Morón[2,8,9], Elmer A. Fernández[1,2,6,10].

[1]ScireLab, Fundación para el Progreso de la Medicina, Córdoba, X5000, Argentina.
[2]Consejo Nacional de Investigaciones Científicas y Técnicas (CONICET), Argentina.
[3]Universidad Tecnológica Nacional. Facultad Regional Córdoba. Córdoba X5000, Argentina.
[4]Fundación Instituto Leloir-CONICET, Buenos Aires, C1405, Argentina.
[5]Research Department, Fundación Huésped, Buenos Aires, C1204, Argentina.
[6]Universidad Argentina de la Empresa (UADE), Instituto de Tecnología (INTEC), Buenos Aires, C1073, Argentina.
[7]Instituto Académico Pedagógico de Ciencias Básicas y Aplicadas, Universidad Nacional de Villa María, Villa María, Córdoba, X5900, Argentina.
[8]Departamento de Bioquímica Clínica, Facultad de Ciencias Químicas, Universidad Nacional de Córdoba (UNC), Córdoba, X5000, Argentina.
[9]Centro de Investigaciones en Bioquímica Clínica e Inmunología (CIBICI), Consejo Nacional de Investigaciones Científicas y Técnicas (CONICET), Córdoba, X5000, Argentina
[10]Facultad de Ciencias Exactas, Físicas y Naturales (FCEFyN), Universidad Nacional de Córdoba (UNC), Córdoba, X5000, Argentina.

Bioinformatic pipeline

The bioinformatic pipeline is run from the R console by using the system2 function. This function allows to run different programs directly onto the linux terminal.

TrimGalore v0.6.6

```
system2(command = “~/TrimGalore-0.6.6/trim_galore”,
        args =c("--paired",
               paste0("--output_dir ",”path/to/output/file”),
               fileR1,
               fileR2)
```

Star v2.7.10b

```
system2(command = “STAR”,
        args = c(paste0("--genomeDir ", “/path/to/STAR_index”),
                paste0("--readFilesIn ",file1, " ", file2),
                paste0("--readFilesCommand ", ifelse(stringr::str_detect(file1,".gz"),"zcat","-")),
                "--runThreadN 8"
                "--outSAMtype BAM Unsorted",
                "--outSAMunmapped Within",
                "--outBAMcompression 0",
                "--outFilterMultimapNmax 50",
                "--peOverlapNbasesMin 10",
                "--alignSplicedMateMapLminOverLmate 0.5",
                "--alignSJstitchMismatchNmax 5 -1 5 5",
                "--chimSegmentMin 10",
                "--chimOutType WithinBAM HardClip",
                "--chimJunctionOverhangMin 10",
                "--chimScoreDropMax 30",
                "--chimScoreJunctionNonGTAG 0",
                "--chimScoreSeparation 1",
                "--chimSegmentReadGapMax 3",
                "--chimMultimapNmax 50")
```

Arriba v2.3.0

```
 system2(command = ”arriba”,
        args = c(paste0("-x ", “/path/to/.bam”),
                paste0("-o ", “fusions.tsv”),
                paste0("-O ", “fusions.discarded.tsv”),
                paste0("-a ", “/path/to/assembly.fa”),
                paste0("-g ", “/path/to/annotation.gtf”),
                paste0("-b ", “/path/to/blacklist.tsv.gz”),
                paste0("-k ", “/path/to/known_fusions.tsv.gz”),
```

```
paste0("-t ", "/path/to/known_fusions.tsv.gz"),
paste0("-p ", "/path/to/protein_domains.gff3"))
```

FeatureCounts

```
FC.object <- Rsubread::featureCounts(files = "/path/to/.bam", annot.inbuilt = "hg38",
juncCounts = TRUE, isPairedEnd = TRUE)
```

Supplementary analysis for: Tumor Fusion Burden is Strongly Correlated with CIN, Impaired Immune Response and Cell Proliferation

Linear models for molecular signatures

Chromosomal instability

```
> summary(lm(TFB ~ CA20+Cohort, TPM2))

Call:
lm(formula = TFB ~ CA20 + Cohort, data = TPM2)

Residuals:
    Min      1Q  Median      3Q     Max
-20.046  -6.378  -2.369   2.490  70.890

Coefficients:
             Estimate Std. Error t value Pr(>|t|)
(Intercept)   -6.6358     4.0315  -1.646 0.101231
CA20           0.7182     0.1815   3.956 0.000104 ***
Cohort067     -3.2895     2.8337  -1.161 0.246984
CohortGide    -0.9817     2.3586  -0.416 0.677668
CohortHugo    10.3800     3.1265   3.320 0.001058 **
CohortMGH      1.7763     3.5087   0.506 0.613202
CohortRiaz    -1.3870     3.1268  -0.444 0.657779
CohortSynder  13.7483     4.9731   2.765 0.006198 **
---
Signif. codes:  0 '***' 0.001 '**' 0.01 '*' 0.05 '.' 0.1 ' ' 1

Residual standard error: 12.18 on 214 degrees of freedom
Multiple R-squared:  0.1957,    Adjusted R-squared:  0.1694
F-statistic: 7.438 on 7 and 214 DF,  p-value: 5.252e-08
```

Cytolytic score

```
> summary(lm(TFB ~ CYT+Cohort, TPM2[-c(78,136),]))

Call:
lm(formula = TFB ~ CYT + Cohort, data = TPM2[-c(78, 136), ])

Residuals:
    Min      1Q  Median      3Q     Max 
-20.964  -6.567  -3.037   2.402  75.545 

Coefficients:
             Estimate Std. Error t value Pr(>|t|)    
(Intercept)  10.44860    2.29338   4.556  8.8e-06 ***
CYT          -0.14136    0.06072  -2.328 0.020861 *  
Cohort067    -4.76731    3.00607  -1.586 0.114253    
CohortGide   -2.64561    2.54544  -1.039 0.299826    
CohortHugo   11.61923    3.18630   3.647 0.000334 ***
CohortMGH     1.52813    3.62904   0.421 0.674120    
CohortRiaz   -0.19023    3.18874  -0.060 0.952486    
CohortSynder 12.66515    5.45588   2.321 0.021216 *  
---
Signif. codes:  0 ‘***’ 0.001 ‘**’ 0.01 ‘*’ 0.05 ‘.’ 0.1 ‘ ’ 1

Residual standard error: 12.48 on 212 degrees of freedom
Multiple R-squared:  0.1492,	Adjusted R-squared:  0.1211 
F-statistic:  5.31 on 7 and 212 DF,  p-value: 1.305e-05
```

Proliferation

```
> summary(lm(TFB ~ Prolif+Cohort, TPM2[-174,]))

Call:
lm(formula = TFB ~ Prolif + Cohort, data = TPM2[-174, ])

Residuals:
    Min      1Q  Median      3Q     Max 
-23.361  -5.634  -2.963   2.168  74.295 

Coefficients:
             Estimate Std. Error t value Pr(>|t|)    
(Intercept)  -2.68777    3.54013  -0.759 0.448556    
Prolif        0.25366    0.07465   3.398 0.000811 ***
Cohort067     1.26090    3.10944   0.406 0.685513    
CohortGide    3.55417    2.69480   1.319 0.188619    
CohortHugo   14.00686    3.14795   4.450 1.39e-05 ***
CohortMGH     5.69871    3.63168   1.569 0.118094    
CohortRiaz    1.22147    3.10146   0.394 0.694095    
CohortSynder 16.96484    5.06660   3.348 0.000961 ***
---
Signif. codes:  0 ‘***’ 0.001 ‘**’ 0.01 ‘*’ 0.05 ‘.’ 0.1 ‘ ’ 1

Residual standard error: 12.27 on 213 degrees of freedom
Multiple R-squared:  0.1866,	Adjusted R-squared:  0.1599 
F-statistic: 6.981 on 7 and 213 DF,  p-value: 1.71e-07
```

Outliers were excluded

Supplementary analysis for: TFB-H is Associated with the Worst ICB Outcome

Univariate Cox regression analysis for clinical data

Cohort

```
> coxph(formula = surv_object ~ Cohort, data = DB_TX)
Call:
coxph(formula = surv_object ~ Cohort, data = DB_TX)

                coef exp(coef) se(coef)     z      p
CohortHugo    0.2087    1.2321   0.3569 0.585 0.5587
CohortMGH     0.3384    1.4027   0.3691 0.917 0.3592
CohortRiaz    0.6692    1.9527   0.3024 2.213 0.0269
CohortSynder 0.8541    2.3492   0.4865 1.756 0.0792

Likelihood ratio test=6.25  on 4 df, p=0.1812
n= 146, number of events= 73
```

Previous treatment

```
> coxph(formula = surv_object ~ previousTX, data = DB_TX)
Call:
coxph(formula = surv_object ~ previousTX, data = DB_TX)

                coef exp(coef) se(coef)     z     p
previousTXyes 0.1202    1.1277   0.2984 0.403 0.687

Likelihood ratio test=0.16  on 1 df, p=0.6908
n= 146, number of events= 73
```

TFB continuous variable

```
> coxph(formula = surv_object ~ logFus, data = DB_TX)
Call:
coxph(formula = surv_object ~ logFus, data = DB_TX)

         coef exp(coef) se(coef)     z       p
logFus 0.2794    1.3223   0.1056 2.646 0.00816

Likelihood ratio test=7.01  on 1 df, p=0.008104
n= 146, number of events= 73
```

Multivariate Cox regression analysis

TFB groups as dichotomous variable

```
> coxph(formula = surv_object ~ group + Cohort + previousTX, data = DB_TX)
Call:
coxph(formula = surv_object ~ group + Cohort + previousTX, data = DB_TX)

                  coef exp(coef) se(coef)      z       p
grouphigh      0.71804   2.05042  0.26916  2.668 0.00764
CohortHugo    -0.05484   0.94664  0.37269 -0.147 0.88302
CohortMGH      0.25502   1.29048  0.38832  0.657 0.51136
CohortRiaz     0.61810   1.85541  0.32299  1.914 0.05566
CohortSynder   0.34294   1.40908  0.52841  0.649 0.51634
previousTXyes  0.31130   1.36521  0.33420  0.932 0.35159

Likelihood ratio test=14.1  on 6 df, p=0.02852
n= 146, number of events= 73
```

TFB as continuous variable

```
Call:
coxph(formula = Surv(OS, V) ~ TFB + Cohort + previousTX, data = as.data.frame(DB_TX))

                   coef exp(coef)  se(coef)      z       p
TFB            0.018443  1.018614  0.007023  2.626 0.00864
CohortHugo    -0.174814  0.839613  0.402131 -0.435 0.66377
CohortMGH      0.386774  1.472224  0.381447  1.014 0.31060
CohortRiaz     0.667582  1.949518  0.318596  2.095 0.03614
CohortSynder   0.524204  1.689114  0.508636  1.031 0.30272
previousTXyes  0.364850  1.440298  0.332062  1.099 0.27188

Likelihood ratio test=13.08  on 6 df, p=0.04186
n= 146, number of events= 73
```

TFB groups as dichotomous variable with ICB regimen and PD-L1 expression

```
Call:
coxph(formula = Surv(OS, V) ~ group + previousTX + TX + PD_L1log,
    data = as.data.frame(DB_TX))

                   coef exp(coef)  se(coef)      z      p
grouphigh      0.567126  1.763193  0.275520  2.058 0.0396
previousTXyes  0.005052  1.005064  0.302393  0.017 0.9867
TXipiPD1      -1.460873  0.232034  0.647843 -2.255 0.0241
TXPD1         -0.125228  0.882296  0.500355 -0.250 0.8024
TXPDL1        -0.499485  0.606843  0.866724 -0.576 0.5644
PD_L1log      -0.392660  0.675258  0.188833 -2.079 0.0376

Likelihood ratio test=24.32  on 6 df, p=0.0004557
n= 144, number of events= 71
   (2 observations deleted due to missingness)
```

Supplementary analysis for: The TFB-H Group is Enriched with M2 Macrophages

Linear model for tumor microenvironment

```
Call:
lm(formula = TFB ~ CA20 + PD_L1log + `Macrophages M2`, data = DB_new)

Residuals:
    Min      1Q  Median      3Q     Max
-15.893  -7.689  -3.230   2.673  78.830

Coefficients:
                 Estimate Std. Error t value Pr(>|t|)
(Intercept)       -9.0127     4.1988  -2.146   0.0329 *
CA20               0.8669     0.1861   4.658 5.56e-06 ***
PD_L1log          -1.4029     1.0730  -1.307   0.1924
`Macrophages M2`   6.8695     4.7145   1.457   0.1465
---
Signif. codes:  0 ‘***’ 0.001 ‘**’ 0.01 ‘*’ 0.05 ‘.’ 0.1 ‘ ’ 1

Residual standard error: 12.74 on 218 degrees of freedom
Multiple R-squared:  0.1025,    Adjusted R-squared:  0.0901
F-statistic: 8.295 on 3 and 218 DF,  p-value: 3.002e-05
```
